# Proglumide Reverses Nonalcoholic Steatohepatitis by Interaction with the Farnesoid X Receptor and Altering the Microbiome

**DOI:** 10.3390/ijms23031899

**Published:** 2022-02-08

**Authors:** Martha D. Gay, Hong Cao, Narayan Shivapurkar, Sivanesan Dakshanamurthy, Bhaskar Kallakury, Robin D. Tucker, John Kwagyan, Jill P. Smith

**Affiliations:** 1 Department of Medicine, Georgetown University, Washington, DC 20007, USA; mdg111@georgetown.edu (M.D.G.); hongc878@gmail.com (H.C.); nms35@georgetown.edu (N.S.); 2 Department of Oncology, Georgetown University, Washington, DC 20057, USA; sd233@georgetown.edu; 3 Department of Pathology, Georgetown University, Washington, DC 20057, USA; kallakub@gunet.georgetown.edu (B.K.); rdt24@georgetown.edu (R.D.T.); 4 Department of Biostatistics, Howard University, Washington, DC 20059, USA; jkwagyan@howard.edu

**Keywords:** nonalcoholic steatohepatitis, microbiome, FXR, CCK receptor, NASH

## Abstract

Nonalcoholic steatohepatitis (NASH) is associated with obesity, metabolic syndrome, and dysbiosis of the gut microbiome. Cholecystokinin (CCK) is released by saturated fats and plays an important role in bile acid secretion. CCK receptors are expressed on cholangiocytes, and CCK-B receptor expression increases in the livers of mice with NASH. The farnesoid X receptor (FXR) is involved in bile acid transport and is a target for novel therapeutics for NASH. The aim of this study was to examine the role of proglumide, a CCK receptor inhibitor, in a murine model of NASH and its interaction at FXR. Mice were fed a choline deficient ethionine (CDE) diet to induce NASH. Some CDE-fed mice received proglumide-treated drinking water. Blood was collected and liver tissues were examined histologically. Proglumide’s interaction at FXR was evaluated by computer modeling, a luciferase reporter assay, and tissue FXR expression. Stool microbiome was analyzed by RNA-Sequencing. CDE-fed mice developed NASH and the effect was prevented by proglumide. Computer modeling demonstrated specific binding of proglumide to FXR. Proglumide binding in the reporter assay was consistent with a partial agonist at the FXR with a mean binding affinity of 215 nM. FXR expression was significantly decreased in livers of CDE-fed mice compared to control livers, and proglumide restored FXR expression to normal levels. Proglumide therapy altered the microbiome signature by increasing beneficial and decreasing harmful bacteria. These data highlight the potential novel mechanisms by which proglumide therapy may improve NASH through interaction with the FXR and consequent alteration of the gut microbiome.

## 1. Introduction

Nonalcoholic steatohepatitis or NASH is a common condition of the liver that is histologically characterized by inflammation, steatosis, fibrosis, and hepatocyte death [1,2]. NASH progresses to cirrhosis in about 20% of the cases [3] and has become a leading risk factor for the development of hepatocellular carcinoma (HCC) [4]. In addition to the obesity epidemic with metabolic syndrome, dysbiosis of the intestinal microbiome has also been implemented in the pathophysiology of NASH [5,6]. Primary bile acids are hydroxylated steroids that are synthesized in the hepatocytes from cholesterol, then conjugated and secreted into the duodenum upon activation of the gastrointestinal peptide cholecystokinin (CCK) [7]. The intestinal microbiome plays an important role in the regulation of bile acid homeostasis and is responsible for converting the primary bile acids to secondary bile acids [8]. The majority of bile acids recirculate to the liver by enterohepatic circulation. Bile acids are the major endogenous ligands for the nuclear farnesoid X receptor (FXR), or NR1H4, a receptor that plays a critical role in regulating bile acid synthesis and enterohepatic circulation [9]. Impaired bile acid signaling is probably the most important mechanism for nonalcoholic fatty liver disease (NAFLD) development [6]. Bile acids in the intestine activate FXR to induce fibroblast growth factor 15 (FGF15) in mice or FGF19 in humans, which in turn are secreted into portal circulation to activate the FGF receptor 4 (FGFR4)/b-Klotho complex located on hepatocytes [10,11]. FGFs are classified as either mitogenic or metabolic, and the endocrine FGF subgroup, including FGF19, 21, and 23, drive metabolic signal axes that elicit broad-spectrum functions in regulating the metabolic homeostasis of bile acid, lipids, glucose, energy, and minerals without direct proliferation-promoting activity [12]. FGFR4 is a tyrosine kinase receptor that upon activation inhibits hepatic cholesterol 7a-hydroxylase (CYP7A1), the key regulatory enzyme in bile acid synthesis [10,11]. In fact, low FGF19 levels have been described in obese subjects with NASH [13]. Although the liver is the predominant location of FGFR4, this receptor is also expressed in the intestine yet at relatively low levels [14]. FXR activation protects against fatty liver injury in animal models of nonalcoholic fatty liver disease and NASH, and improves hyperlipidemia, glucose intolerance, and insulin sensitivity [15]. Consequently, FXR has become a target for new drug development for the treatment of NASH, since activation of FXR increases bile flow thereby improving liver histology.

Cholecystokinin (CCK) receptors play an essential role in fatty acid absorption and bile secretion. Cholangiocytes have been shown to express CCK-B receptors [16,17] that respond to gastrin or CCK by decreasing bile secretion. CCK peptide is released from the duodenal I-cells in response to dietary fat [18] and this peptide acts with equal affinity as gastrin at the CCK-B receptor. CCK also stimulates gallbladder contraction by activation of the CCK-A receptor. Proglumide is a nonselective CCK-A and CCK-B receptor inhibitor [19] with a chemical structure (DL-4-benzamido-N, N-dipropylglutaramic acid) that resembles that of bile acids and could possibly mediate its effects by interacting with the FXR. In rodents, proglumide increases bile flow [20] and decreases bile acid concentration. We previously showed that proglumide therapy prevented and reversed biochemical and histologic NASH in mice fed a choline deficient ethionine (CDE) diet [21]. In this model, we found that proglumide significantly reduced serum bilirubin levels, suggesting that proglumide was stimulating bile flow and reversing hepatocyte injury. In this investigation, we studied the interaction of proglumide with FXR in the liver and its role in altering the intestinal microbiome in CDE-fed mice with confirmed NASH.

## 2. Results

### 2.1. Effects of CDE Diet and Proglumide on Food Intake and Body Weight

**Figure 1:**
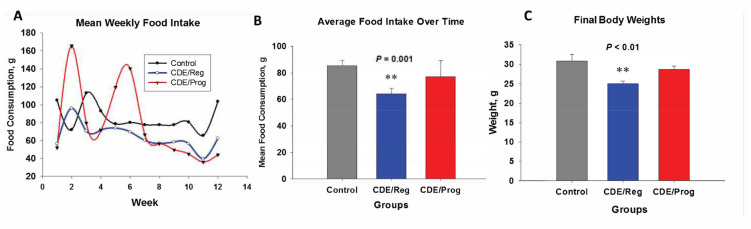
Effects of CDE diet and proglumide on food intake and body weight. (**A**) Weight of food in grams consumed by each group weekly. (**B**) Mean ± SEM of food intake during the study. Significantly different from control and CDE/Prog groups ** *p* = 0.001. (**C**) Final body weights (mean ± SEM) for each group at the termination of the study. Significantly different from control and CDE/Prog groups ** *p* < 0.01. (**A**,**B**) modified from reference [21] with permission.

Because proglumide may block CCK receptors in the brain and negatively affect satiety [22], weekly food intake for each treatment group and controls was measured over 12 weeks (Figure 1A). The mean food intake in grams for each group over time showed that mice in the CDE/Reg group consumed significantly less food than the control mice (Figure 1B). Proglumide-treated mice on the CDE diet consumed similar food quantity as the control mice (Figure 1B). Since the CDE/Reg–fed mice consumed less food, their final body weights were significantly less than control and CDE/proglumide-fed mice (Figure 1C). These data would suggest that proglumide affected food intake.

### 2.2. Proglumide Ameliorates the Effects of NASH Biochemically and Histologically

Confirmation of biochemical NASH was determined by evaluating serum transaminases in CDE-fed mice compared to control mice at the time of euthanasia (week 18). Aspartate aminotransferase (AST), alanine aminotransferase (ALT) and total bilirubin were elevated in mice fed the CDE diet when compared to control mice on standard chow (Table 1), and these values were significantly reduced in CDE-fed mice by the addition of proglumide to the drinking water (CDE/Prog). Histologic analysis confirmed that proglumide therapy significantly decreased the inflammation, fibrosis, and steatosis scores compared to mice fed the CDE diet with untreated water (Table 1).

**Figure 2:**
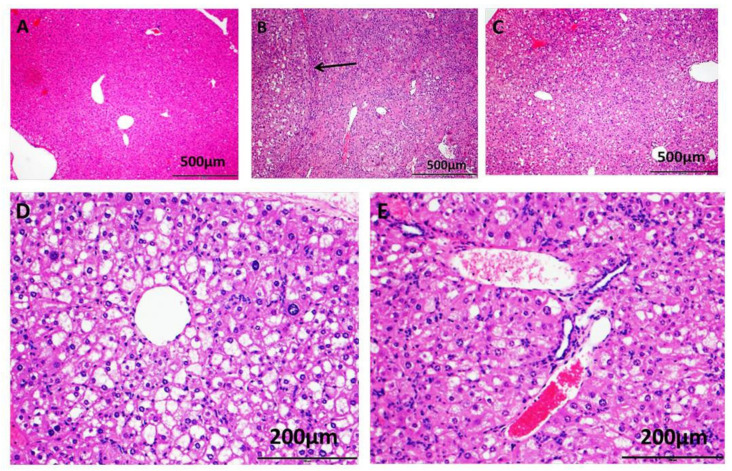
Representative H&E-stained liver tissues from mouse groups. (**A**) H&E section from the liver of a control mouse shows normal hepatic histology, scale 500 μm. (**B**) H&E image from a mouse in the CDE/Reg group reveals steatosis, inflammation, and fibrosis, scale 500 μm. Hepatocellular carcinoma developed in this mouse after 18 weeks on the CDE diet (arrow). (**C**) H&E image from the liver of a mouse fed the CDE diet for 18 weeks but also treated with proglumide (CDE/Prog) shows some steatosis but less inflammation and fibrosis compared to the liver of the CDE/Reg mouse, scale 500 μm. (**D**) Histologic section from the liver of a CDE/Reg mouse shows typical features of NASH in this perivenular area of the liver including macro- and micro-steatosis, inflammation, fibrosis and balloon degeneration, scale 200 μm. (**E**) Image from the liver of a CDE/Prog-fed mouse shows less inflammation, steatosis, and fibrosis in the liver, scale 200 μm.

Representative histologic sections of H&E-stained livers from each group are shown in Figure 2. Liver histology from a control mouse is shown (Figure 2A) compared to that of a liver from a CDE/Reg-treated mouse (Figure 2B) and a liver from a mouse fed the CDE diet with proglumide (CDE/Prog) (Figure 2C). Mice on the CDE diet exhibited the characteristic features of NASH with histologic evidence of steatosis, inflammation, and fibrosis. In addition, some mice on the CDE/Reg diet developed hepatocellular cancer or dysplastic nodules at week 18 (Figure 2B, arrow). A higher magnification (10×) shows a representative liver tissue section from a mouse from the CDE/Reg cohort with inflammation, steatosis, fibrosis, and balloon degeneration (Figure 2D). A representative image taken at the same magnification from a liver section of a mouse fed CDE/Prog shows some steatosis and less inflammation and fibrosis (Figure 2E) as compared with the CDE/Reg mouse liver. These results confirm that histologic and biochemical NASH was induced in the mice fed the CDE diet for 18 weeks and that proglumide ameliorated the histologic effects of this diet.

### 2.3. Effects of Proglumide on Differentially Expressed Genes (DEGs)

**Figure 3:**
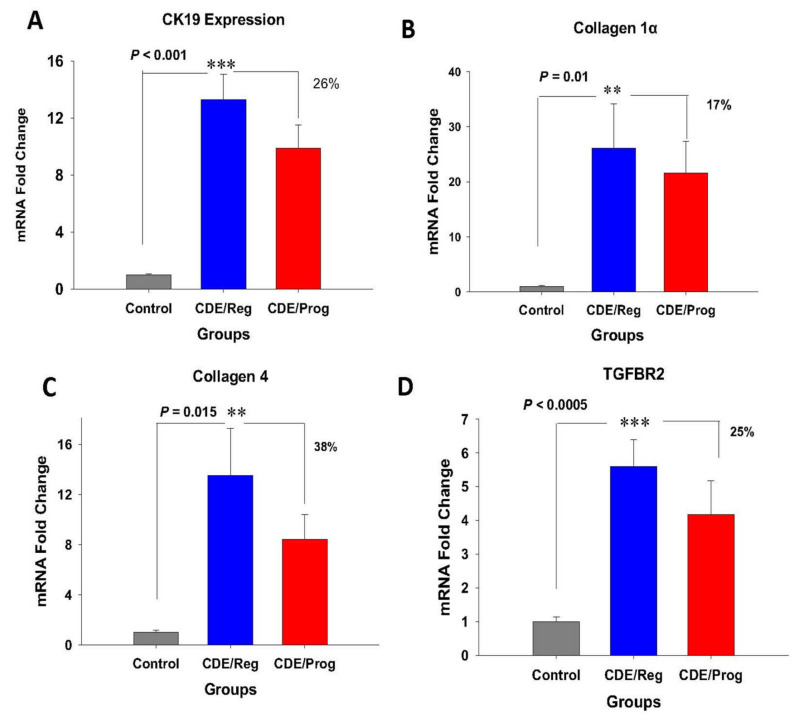
Effects of CDE diet and proglumide on genes involved in proliferation and fibrosis of the liver. Mean ± SEM of mRNA expression for (**A**) *CK19*, (**B**) *collagen-1α*, (**C**) *collagen-4,* and (**D**) *TGBβR2* were all significantly increased in the livers of CDE-fed mice by qRT-PCR (blue columns). Proglumide decreased the mRNA expression of these genes while mice were on the CDE diet (red column). Significantly different from livers of control mice ** *p* = 0.015 and *** *p* < 0.0005.

Several differentially expressed genes (DEGs) were identified that were significantly upregulated in the livers of mice with NASH from the CDE diet, and treatment with proglumide decreased the expression of these genes. Cytokeratin 19 (CK19), a marker of oval/progenitor cell proliferation [23], is elevated in NASH livers [24]. The livers of mice fed the CDE/Reg diet expressed high levels of CK-19 compared to control mice, and this effect was ameliorated by proglumide (Figure 3A). Collagen-1α (Figure 3B) and collagen-4 (Figure 3C) were both significantly elevated in the livers of mice on the CDE diet and represent the increased fibrosis identified in these livers. Proglumide therapy decreased both of the collagen-associated genes while continuing the CDE diet. TGFβ signaling is an important pathway leading to increased extracellular matrix and is associated with increased expression of TGFβ-receptor-2 (TGFβR2). CDE-fed mice had significantly increased expression of TGFβR2 (Figure 3D), and the expression of this gene was decreased in livers of mice treated with proglumide.

**Figure 4:**
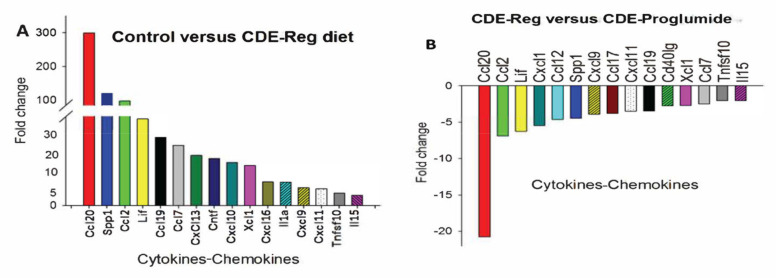
Expression of liver chemokines and cytokines in CDE-fed mouse livers compared to controls. (**A**) mRNA expression of several cytokines and chemokines are significantly increased in livers of CDE-fed mice with NASH compared to control mice with normal liver histology. (**B**) Chemokine and cytokine mRNA expression in livers of CDE-fed mice are reversed with proglumide therapy. (The figure was reproduced with the permission from Rightslink^®^, published in Cancer Prevention research [27].)

Since one of the major histologic characteristics of NASH is inflammation, we performed a cytokine/chemokine PCR array to study changes in inflammatory genes in the NASH livers that were altered by proglumide. As expected, mRNA expression of several cytokines/chemokines was significantly increased in the NASH livers compared to controls (Figure 4A) and proglumide treatment significantly decreased the expression of these chemokines (Figure 4B). Most of these cytokines and chemokines are involved in macrophage recruitment. One chemokine in particular, *CCL20* (also called Macrophage Inflammatory Protein-3), was increased in the livers of NASH mice and this level was reduced greater than 20-fold with proglumide. *CCL20* has been implemented as a proangiogenic factor in hepatitis C-associated HCC [25]. Another cytokine, *CCL2*, has been shown to play an important role in the regulation of HCC tumor growth, metastasis, and host immune response [26]. *CCL2* was also significantly reversed with proglumide.

### 2.4. Computational Ligand Modeling Reveals Binding of Proglumide to FXR

**Figure 5:**
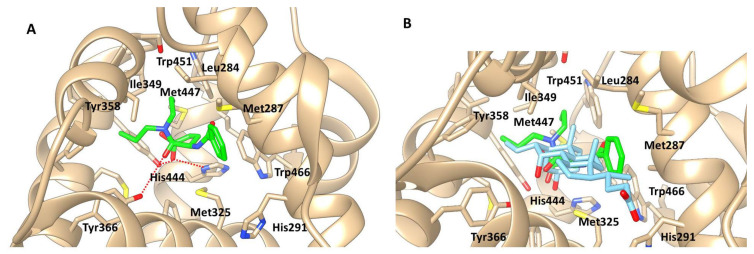
Computer modeling of proglumide and the FXR. (**A**) Magnified view of structural model proglumide complex with FXR. The FXR amino acids interacting with proglumide are shown as stick model. Hydrogen bonds are shown in broken lines (red). Proglumide carbon atoms are colored green. (**B**) Image of a bile acid superimposed with the structural model of proglumide complex with FXR. The FXR amino acids interacting with proglumide are shown as stick model. Proglumide carbon atoms are colored green. Bile acid carbon atoms are colored in magenta.

Computerized drug target prediction and ligand-based modeling programs have become powerful tools to facilitate the understanding of pharmacokinetic and pharmacodynamic properties of ligands with their targets [28]. Computer modeling with the chemical structure of proglumide demonstrates interaction with the FXR (Figure 5A). When computational binding is performed with the natural ligand for FXR, a bile acid, binding is easily superimposed upon proglumide (Figure 5B). These data suggest that proglumide may also serve as a ligand for FXR.

### 2.5. Proglumide Interacts with FXR as a Partial Agonist

**Figure 6:**
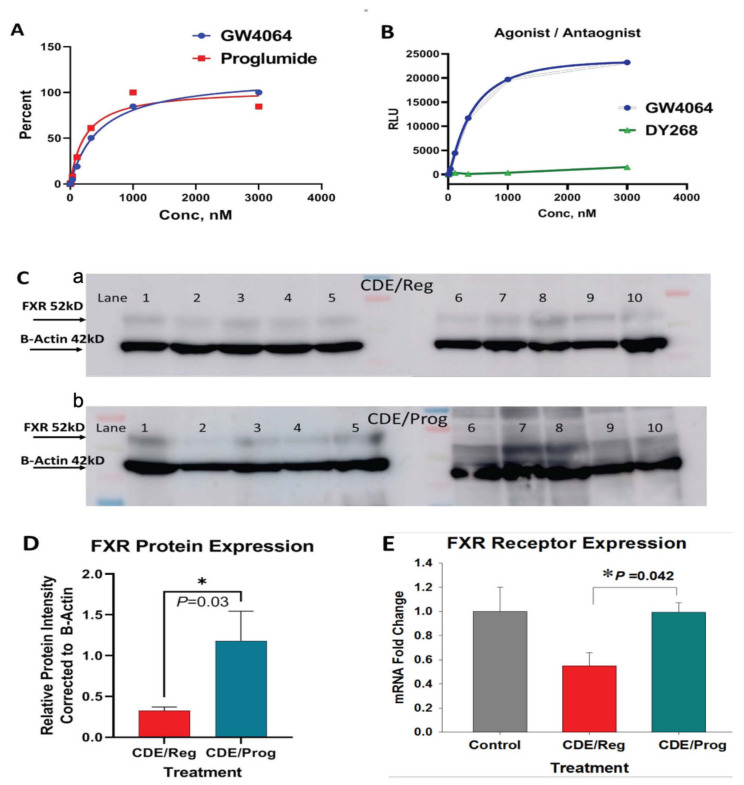
Proglumide’s interaction with FXR. (**A**) Results of proglumide interaction at the FXR as evaluated by the FXR reporter assay shows the expected sigmoidal curve of the FXR agonist, GW4064, with an EC_50_~312.7 nM. Proglumide reacts with the FXR receptor with a characteristic agonist curve similar to GW4064 and an EC_50_~214.9 nM. (**B**) Results of the reporter assay showing characteristic plot with the agonist (GW1464) compared to that of the FXR antagonist DY268. (**C-a**) FXR protein expression by Western blot is shown for NASH livers of mice on CDE/Reg diet (a) *N* = 10) and from livers of mice on CDE/Prog diet ((**C-b**), *N* = 10)). Protein expression is normalized with β-actin. (**D**) Densitometry analysis of the Western blot above for FXR protein expression is analyzed and plotted as a ratio normalized to β-actin. FXR expression is significantly increased in the mice on the CDE diet treated with proglumide compared to mice on the CDE diet with untreated water (* *p* = 0.03). (**E**) FXR mRNA expression as measured by qRT-PCR shows a decrease in the FXR expression in the CDE/Reg-fed mouse livers compared to FXR expression in the normal mouse liver. Restoration of the mRNA levels to control levels is shown in the livers of mice treated with proglumide (* *p* = 0.042).

Using the FXR reporter assay, proglumide’s ability to interact with the FXR was tested over a wide range of concentrations. Proglumide exhibited agonist binding properties similar to the agonist GW4064 (Figure 6A) and had a mean EC_50_ of 215 nM (range 134.8 to 343.2 nM). The EC_50_ for GW4064 in the reporter assay was determined at 312.7 nM (range 221.7 to 441.2 nM). Since there was little difference in the EC_50_ between proglumide and the known FXR agonist GW4064, these findings suggest that proglumide acts as a partial agonist at the FXR receptor. The results of the competition binding reporter assay for the FXR antagonist DY268 shows a typical inhibition binding curve (Figure 6B). Since the reporter assay binding curve for proglumide parallels that of the agonist GW4064 rather than the antagonist DY268, we conclude that proglumide interacts with FXR with properties consistent with an agonist.

### 2.6. Proglumide Treatment Restores FXR Expression in Tissues of CDE-Fed Mice

The expression of FXR and FGFR4 are downregulated during NASH development [15] and FXR-deficient mice develop NASH-associated liver damage [29]. FXR protein expression was compared in the livers of mice fed the CDE/Reg diet to the CDE/Prog diet by Western blot (Figure 6C). Densitometry of Western blots were normalized to β-actin and revealed that FXR protein expression was significantly increased in the livers of mice in the CDE/Prog group (Figure 6D; *p* = 0.03). Liver FXR mRNA expression was also evaluated by qRT-PCR and found to be downregulated in the mice on the CDE/Reg diet compared to untreated control mice (Figure 6E). CDE-fed mice that were concomitantly treated with proglumide showed increased mRNA expression (*p* = 0.042) for FXR in the liver tissues with levels similar to those of the control mouse livers (Figure 6E). Overall, these results confirm that proglumide may have partial agonist activity at FXR, FXR expression is downregulated in NASH livers, and proglumide restores the expression to normal levels.

### 2.7. Proglumide Treatment Restores FGFR4 Expression in Tissues of CDE-Fed Mice

FGFR4 protein expression was evaluated in the livers of CDE-fed mice and was downregulated by Western blot (Supplementary Materials; Figure S1A) compared to that of FGFR4 expression in a control/normal mouse liver. CDE-fed mice that received proglumide exhibited increased FGFR4 expression in liver protein extracts compared to CDE-fed mice with untreated water (Supplementary Materials; Figure S1A). Densitometry of the Western blot bands for FGFR4 expression was normalized to actin and analysis showed that CDE/Prog mice had significantly increased expression of FGFR4 (Supplementary Materials; Figure S1B; *p* = 0.01). FGFR4 protein expression was also evaluated by Western blot in the intestines from mice on standard chow receiving untreated drinking water or proglumide-treated water (Supplementary Materials; Figure S2A). mFGFR4 expression was analyzed by densitometry and was found to be increased 3-fold in intestines of mice treated with proglumide compared to control mice, but this did not reach statistical significance (*p* = 0.06; Supplementary Materials; Figure S2B). Perhaps the results of FGFR4 expression in the intestine did not reach statistical significance because this cohort of mice was treated with proglumide for 6 weeks rather than 18 weeks because FGFR4 expression is significantly lower in the intestine than in the liver [30]. However, the 3-fold increase in FGFR4 expression in the intestine for the proglumide-treated mice suggests that proglumide may also alter the intestinal FGFR4 axis. Overall, these results confirm that proglumide treatment alters expression of the FXR-FGFR4 axis in mouse tissues, FGFR4 expression is downregulated in NASH livers, and proglumide restores the expression to normal levels.

### 2.8. Proglumide Therapy Alters the Gut Microbiome

**Figure 7:**
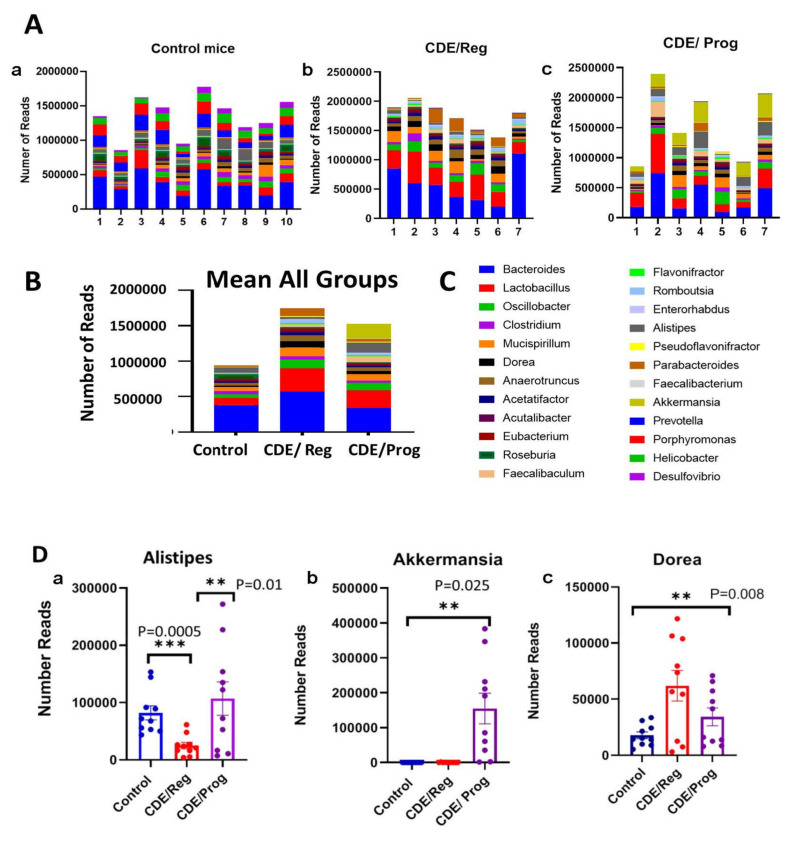
Microbiome analysis by RNAseq in control mice compared to that of CDE/Reg and CDE/Prog mice. (**A**) The number of reads for the top 24 genera represented in stacked columns with each color representing a different genus and the height of the color represents the number of reads for that genus. The individual results for each mouse are shown for the control mice (*N* = 10 (**A-a**)), the CDE/Reg mice (*N* = 7, (**A-b**)), and the CDE/Prog mice (*N* = 7, (**A-c**)). (**B**) The mean number of reads in stacked columns for each group is shown with an increase in pathogenic bacteria (i.e., Bacteroides) in the CDE/Reg mice compared to controls and this level is decreased in mice in the CDE/Prog group. (**C**) A list of the represented genus by name with color coding is shown and correlates with (**A**,**B**). (**D**) The number of individual reads and mean ± SEM of three different bacteria (Alistipes, Akkermansia, and Dorea) that were specifically altered in the microbiome of mice with NASH on the CDE/Reg diet. In mice on the CDE/Prog diet, the beneficial bacteria Alistipes (** *p* = 0.01, *** *p* = 0.0005) and Akkermansia (** *p* = 0.025) increase in the number of reads while the pro-inflammatory bacteria Dorea decreases with proglumide therapy (** *p* = 0.008).

Fecal samples were collected from the 18-week-old mice and subjected to whole genera 16s sequencing with analysis of bacteria genera for the number of reads and the diversity. The top 24 genera are shown (Figure 7A) for each cohort in stacked columns with the magnitude representing the abundance of each particular genus. Individual specimens are expressed for each cohort on the top panel including the control mice (Figure 7A) CDE/Reg mice, and the CDE/Prog mice. In the lower panel of Figure 7B the mean values of all the independent samples are shown, and the color panel with coding for each genus to the right in Figure 7C. Bacteroides is the most abundant bacteria (blue coloring on the bottom of the columns), and the amount of Bacteroides increases with the development of NASH but decreases with proglumide therapy. The increase in these pathogenic bacteria with the development of NASH and fibrosis has been well described in animal models and human subjects [31,32].

Several beneficial bacteria were found to be significantly increased in the feces of mice treated with proglumide. Alistipes is a fairly new genus that is classified as a Gram-negative, rod-shaped, anaerobic, and non-spore forming organism; this organism was significantly increased in the microbiome of proglumide-treated mice (Figure 7D-a). In relationship to fatty liver disease, Alistipes has been shown as protective against the development of fibrosis [33], consistent with the finding that the livers of the CDE/Prog mice had significantly less fibrosis. Another beneficial bacteria that was significantly increased with proglumide therapy was Akkermansia (Figure 7D-b). The bacteria Akkermansia (*A. muciniphila*) belongs to the phylum *Verrucomicrobia* and is a mucosal-associated bacteria related to the health of the gut microbiome [34]. Recent studies have shown that Akkermansia is decreased in human subjects with metabolic syndrome and NAFLD and that dietary supplementation with this organism in obese individuals can improve hepatic transaminases and blood glucose [35,36]. The bacteria Dorea increases in abundance in the mice fed the CDE/Reg diet that and had histologic NASH (Figure 7D-c) compared to control mice; this is reduced in mice on the CDE/Prog treated diet. Dorea is characterized in the phylum Firmicutes and in the family of Lachnospiraceae. This bacterium is increased in the gut microbiome of those with NAFLD compared to healthy controls [37,38]. An increase of Dorea in the gut bacteria has been identified as a typical “microbiota signature” associated with NAFLD-NASH progression [39].

## 3. Discussion

This study showed that a cholecystokinin receptor inhibitor, proglumide, decreases the histologic and biochemical development of NASH in a murine model. One mechanism of action to explain this finding was previously attributed to the interaction of proglumide at the CCK-B receptors that are increased in expression in the mouse NASH liver [27]. However, a novel finding of this investigation is that proglumide also decreases NASH by acting as a partial agonist at the farnesoid X receptor in the murine liver. We demonstrated by computer modeling and with FXR reporter binding assays that proglumide selectively interacts with FXR in a fashion similar to that of bile acids and the known FXR agonist GW4064. Expression of FXR and FGFR4 were decreased in the livers of mice with confirmed NASH when compared to normal mouse liver, and these levels were restored to normal in mice on the same saturated fat diet when treated with proglumide. Another unique finding from the present investigation was that proglumide also altered the gut microbiome in mice, changing the bacterial signature to one more comparable to that of the healthy control mice.

Bile acids have been shown to play an important role in the treatment of NASH by interactions at the FXR. One bile acid, 6-ethylchenodeoxycholic acid, or obeticholic acid (OCA), has been shown in preclinical murine models of NASH to decrease fibrosis and improve hepatic histology [40,41]. Clinical trials in human subjects with NASH have been conducted with OCA [42,43]; however, a Phase 3 clinical trial fell short of achieving the major endpoints necessary to gain FDA approval. In addition, a significant number of the subjects treated with OCA developed pruritus as a common side effect. Proglumide has a chemical structure very similar to that of bile acids and is described as an N(2)-benzoyl-N,N-dipropyl-alpha-glutamine with a molecular weight of 334.4 g/mol [44]. A distinctive characteristic of proglumide not found in other CCK receptor antagonists is that proglumide has been shown to increase bile flow [20] and decrease bile acid concentration in animal models by an “unknown mechanism of action.” Based on our results, we suggest that proglumide may alter bile flow and bile acid concentration by its interaction with the FXR-FGFR4 axis. Obeticholic acid is a synthetic bile acid derivative with an EC_50_~99 nM for the FXR [45]. In contrast, the dose–response analysis for naturally occurring bile acids indicated that chenodeoxycholic acid (CDCA), deoxycholic acid (DCA), and lithocholic acid (LCA) display an EC_50_ of approximately 50 μM [46]. Hence, OCA is about a 100-fold more potent agonist at FXR than the naturally occurring bile acids [47]. Our binding assays confirm that proglumide interacts with FXR as an agonist with similar potency to GW4064. Since the EC_50_ of proglumide is less potent than OCA, this compound may have a safer and less toxic side effect profile than OCA.

The expression of FXR and FGFR4 are also downregulated during NASH development [15], and FXR activation is protective against liver inflammation associated with NASH. FXR deficiency (i.e., FXR-knockout mice) is associated with progression and/or exacerbation of NASH [48]. We confirmed that FXR and FGFR4 mRNA expression were downregulated in the livers of our mice with histologically confirmed NASH, and the expression of these bile regulating receptors was reversed in mice treated with proglumide. These results substantiate the interaction of proglumide at the FXR signaling pathway, thus supporting the conclusion that the protective liver findings of mice treated with proglumide are associated with FXR expression.

Recently, dysbiosis of the gut microbiome has been associated with several medical conditions, including NASH [49,50,51,52]. Furthermore, fecal, or intestinal microbiota transplantation improves hepatic function in those with NASH and/or cirrhosis [53]. Analysis of the gut microbiome from those with NASH typically show a decrease in bacterial diversity [31] and an increase in pathogenic bacteria such as Bacteroides as was found in our study. Often, Firmicutes are thought to represent beneficial bacteria and are usually decreased in NASH when analyses are performed at the phylum level. However, some studies have shown [31] that deep sequencing of gut microbiome with analysis at the genus level (rather than the phylum level) reveal that certain pathogenic Firmicutes are increased such as those in the Lachnospiraceae genus (*Robinsoniella, Roseburia,* and *Dorea*) and certain *Lactobacillus.* We also found that some pathogenic Firmicutes such as Dorea and Lactobacillus were increased in mice with NASH. These results imply the potential disadvantage of using higher phylogenetic levels (i.e., phylum) to distinguish disease states [31]. An interesting finding from our investigation was that proglumide therapy altered the gut microbiome in mice, changing its bacterial signature to that of a more normal flora than pathogenic. In particular, some beneficial bacteria that were significantly increased in the microbiome of mice treated with proglumide were identified, including Alistipes and Akkermansia. A fecal decline in Alistipes has been associated with worsening hepatic fibrosis and hepatic encephalopathy [54]. The abundance of Akkermansia has been inversely correlated to body fat mass and glucose intolerance in mice, but more evidence is needed in humans. In a randomized placebo controlled clinical trial, oral administration of *Akkermansia muciniphila* reduced the levels of the relevant blood markers for liver dysfunction and inflammation [36].

The mechanism by which proglumide alters the gut microbiome may be related to the effects of proglumide on the bile acids. Bile acids have both direct antimicrobial effects on gut microbes, and indirect effects through FXR-induced antimicrobial peptides [8]; proglumide has been shown to affect bile acid concentration and secretion [20]. Other possible explanations for proglumide’s effects on the microbiome include changes in gut motility or decrease in gastric acid secretion through antagonism at the CCK-B receptor. Since proglumide is a nonselective CCK receptor antagonist [55] it can interfere with activation of both the CCK-A and CCK-B receptors. Proglumide may also interact with the TGR5 receptor (or GP-BAR1, or M-BAR). The TGR5 receptor was characterized years ago as the first G-coupled protein receptor specific for bile acids [56], and TGR5 is expressed in mice within the gallbladder, bile ducts, Kupffer cells, and intestine [57]. Recently, the TGR5 receptor has been found to co-localize with the CCK-A receptor and deoxycholic acid (a bile acid), and CCK treatment exhibited a synergistic effect on satiety [58].

CCK receptors have been characterized on stellate cells of the pancreas [59] and on fibroblasts [60] and stimulation of these receptors results in collagen deposition and fibrosis. We have previously shown that proglumide therapy could decrease fibrosis by preventing stellate cell activation in the mouse liver [21] and in the pancreas microenvironment [61]; therefore, we assume that the anti-fibrotic effect seen in the livers of the CDE-fed mice treated with proglumide in this investigation was related to the blockade of the CCK receptor on mouse stellate cells in the liver. The reduction in hepatic inflammation observed in the CDE/Prog-treated mice of this study may be due to proglumide’s action on reducing inflammatory cytokines and chemokines as previously described [27]. The mechanism by which proglumide decreases cytokines and inflammation is unknown; however, CCK receptors and chemokine receptors are both G-protein coupled receptors (GPCRs). GPCRs are known to “cross-talk” or influence the action of the other GPCRs, either by sensitizing or desensitizing the intracellular signaling or downstream pathways or by regulating the actions by forming “heterodimers” [62]. It is possible that proglumide cross-talks with chemokine receptors to downregulate inflammation.

## 4. Materials and Methods

### 4.1. Animal Model

All mouse studies were performed in an ethical fashion and approved by the Institutional Animal Care and Use Committee at Georgetown University. Thirty female C57BL/6 mice (Charles Rivers) ages 6–8 weeks were randomized to one of three groups (*N* = 10 per group). The control group received standard chow and untreated drinking water. One group was fed a 75% choline deficient ethionine supplemented (CDE) diet (MP Biomedicals, Solon, OH, USA) with regular, untreated drinking water (CDE/Reg). The third group (CDE/Prog) was fed the same 75% CDE diet but received drinking water treated with proglumide (0.1 mg/mL), a concentration we previously demonstrated blocked the CCK receptor in mice [61]. Casein was the major source of protein and lard (saturated fat) was the primary source of fat in this diet. Food intake was measured weekly for 12 weeks and final body weights recorded at the time of euthanasia. At week 18, the mice were ethically euthanized, blood collected and livers were excised for histology. Evidence of biochemical NASH was confirmed with the determination of serum hepatic transaminases and bilirubin. RNA was extracted from livers and analyzed for differentially expressed genes by qRT-PCR. Primers used for the mRNA expression are shown in the Supplementary Materials Table S1. Liver tissues were fixed, paraffin embedded, and 5 µm tissue sections on slides stained with hematoxylin and eosin (H&E). Using the criteria in our previously published study [21], histologic NASH was scored for inflammation, fibrosis, and steatosis by a pathologist blinded to the treatment groups.

Another group of C57BL/6 mice that were fed standard chow were analyzed to determine if proglumide altered FGFR4 expression in the mouse intestine. Mice were provided untreated water (*N* = 10) or proglumide-treated water (*N* = 10) for six weeks. Mice were ethically euthanized and small intestines excised and subjected to analysis by Western blot for FGFR4 expression.

### 4.2. Molecular Modeling and Docking Studies

The X-ray crystal structure of the FXR complexed with bile acid (Protein Data Bank code: 1OSV) was used for the molecular docking simulations of proglumide. FXR structure was minimized with AMBER with SANDER default parameters [63]. Docking experiments were performed using Surflex Dock module of Sybyl X-2.2 (Certara, Princeton, NJ, USA), with the number of solution conformations set to 90. The best docked geometry of proglumide was visually analyzed, and energy-minimized with the SANDER module of AMBER [63].

### 4.3. Molecular Dynamics Simulations

To obtain a final structural model of the FXR–proglumide complex, the minimized was further relaxed to include flexibility of the protein and ligand. The flexibility was included by performing molecular dynamics simulations using the AMBER simulation package [63] with the general amber force field [64] and root-mean-square deviation charge models [65]. Molecular dynamics simulations performed in the NVE (number of atoms/volume/energy) ensemble consisted of an initial equilibration of 100 ps followed by a production run of 500 ps dynamics at 300 K. The final complex structure at the end of the molecular dynamics simulation was subjected to 5000 steps of steepest descent energy minimization followed by conjugate gradient energy minimization. Distance-dependent dielectric constant and nonbonded distance cutoff of 12 Å were used.

### 4.4. Human FXR Luciferase Reporter Assay

A human Farnesoid X Receptor (FXR) Reporter Assay kit was purchased from INDIGO Biosciences, Inc. (Indigo Biosciences, State College, PA, USA, Cat # IB00601). The FXR agonist GW4064 was prepared for 2× its concentration and added to reporter cells before dispensing into a 96-well plate with a total of 100 µL/well. Cells were then placed in the incubator for up to 3 h to adhere before using compound screening media to prepare serial dilutions (1 mM, 500 µM, 250 µM, 125 µM, 62.5 µM, 1.25 µM, 0.125 µM) of proglumide. Reporter cells were combined with 2x GW4064, the reference agonist, and then dispensed in 100 µL in all wells. Next, 100 µL of 2× concentration of DY268, (Tocris, Bristol, UK, Cat # 1609564-75-1) the antagonist, was dispensed in wells designated with the GW4064, the agonist. The plate was incubated overnight at 37 °C and the next day luciferase detection reagent (100 μL) was added and the luminescence was evaluated using a BMG LabTech, Model: FLU Ostar Omega.

### 4.5. FXR and FGFR4 Hepatic Expression by Western Blot

Protein was extracted from mouse liver and intestine with the RIPA buffer plus proteinase inhibitor mini tablet (ThermoFisher, Waltham, MA, USA, Cat # A32955). Extracts were boiled with 4X NuPAGE LDS Sample Buffer (Invitrogen, Waltham, MA, USA, Cat # NP0007) at a 4:1 ratio. Samples of equal protein (100 µg) were loaded onto NuPAGE 4–12% Bis-Tris gels (Invitrogen, Waltham, MA, USA, Cat # NP0321BOX), separated by electrophoresis at voltage 200, and then transferred to nitrocellulose membrane (ThermoFisher, Waltham, MA, USA, Cat # 88018) at voltage 50 for 2 h. The membrane was blocked in 5% non-fat milk for 1 h at RT and then probed with a rabbit anti-FGFR4 polyclonal antibody (Abcam, Cambridge, UK, Cat # 5481) at a dilution of 1:1000 overnight. Similarly, protein lysates (40 μg) from livers of CDE/Reg mice (*N* = 10) or CDE/Prog mice (*N* = 10) were analyzed by Western blot using a rabbit polyclonal antibody to FXR or anti-NR1H4 (Abcam, Cambridge, UK, Cat #85606) at a dilution of 1:100 overnight. The blots were then incubated with anti-rabbit IgG at RT for 1 h, conjugated to horseradish peroxidase, and developed by Western Bright ECL-Spray (Advansta, San Jose, CA, USA, Cat # K-12049-D50). The Spectra Multicolor Broad Range Protein Ladder (ThermoFisher, Waltham, MA, USA, Cat # 26634) was used for molecular weight determinations. For loading normalization, the same membrane was blotted with a mouse monoclonal anti-β actin antibody (Invitrogen, Waltham, MA, USA, Cat # MA1-140) at a dilution of 1:1000 overnight, followed by incubation with an anti-mouse IgG at RT for 1 h, and developed by ECL. The densitometry of the bands was quantified using image-J software.

### 4.6. Hepatic FXR mRNA Expression

Total RNA was extracted from three different groups of mouse livers (*N* = 3 each): (i.) CDE-fed mice for 18 weeks with confirmed NASH histologically; (ii.) CDE-fed mice for 18 weeks that received concomitant proglumide; and (iii.) from mice fed standard chow and untreated water using a RNeasy plus Mini Kit (Qiagen, Germantown, MD, USA, Cat # 74134) to evaluate mRNA expression of FXR genes. Complementary DNA was generated and subjected to real-time PCR (qRT-PCR) using SYBR^®^ Green (Quanta Biosciences, Beverly, MA, USA, Cat # 95073-012) in an Applied Biosystems 7300 thermal cycler with the following conditions: initial incubation for 3 min at 95 °C followed by 40 cycles of 15 s at 95 °C, and extension 1 min at 60 °C using FXR mouse primers with the sequences Forward: 5′TGTGAGGGCTGCAAAGGTT3′ and Reverse: 5′ACATCCCCATCTTGGAC 3′.

### 4.7. Microbiome Analysis

High-throughput sequencing (HTS) was performed on stool pellets collected from individual mice from those fed the CDE diet with regular water (*N* = 7), the CDE diet with proglumide (*N* = 7), and control chow (*N* = 10). All the mice were 18 weeks of age when the samples were collected when the CDE/fed mice had established NASH. Fecal DNA extraction was performed using the Qiagen DNeasy 96 PowerSoil Pro QIAcube HT extraction kit according to the manufacturer’s protocol. Library preparation was performed using the KAPA HyperPlus library preparation protocol. Whole genome 16s sequencing (WGS) was performed using the Illumina NovaSeq 6000 instrument by Transnetyx, Inc. (Cordova, TN, USA). Data were processed on the One Codex platform [66] according to the instructions outlined using two reference databases. The One Codex identifies microbial sequences using a reference database that currently includes approximately 40,000 bacterial, viral, fungal, and protozoan genomes [66] and a smaller database containing the over 8000 microbial genomes contained in the NCBI RefSeq database.

### 4.8. Statistical Analysis

All data were analyzed using GraphPad version 9 (PRISM). Analysis of biochemical and molecular differences between the control mice and the mice on the CDE diet was performed with Student’s *t-*test. One-Way ANOVA was used to compare mean FXR mRNA and protein expression levels among the three groups (the CDE/Reg, CDE/Prog and control). When the *F*-test from ANOVA indicated significance, the Bonferroni test was used for pairwise comparison. The molecular and docking studies of proglumide were analyzed with the SANDER module of AMBER [63]. Analysis of agonist dose–response binding assay was performed using PRISM to generate dose–response curves and to calculate the EC_50_ for proglumide compared to the known agonist GW4064. The mean relative light units (RLUs) from the data were normalized to percent of response such that the smallest data point in each set was defined at 0% and the largest data point in each set was defined as 100%. The equation utilized from PRISM to calculate the EC_50_ was generated from the “dose–response agonist” selection. Species differences in the gut microbiome between the CDE/Reg and CDE/Prog groups were analyzed by calculating the mean of each group and comparison by Student’s *t*-Test.

## 5. Conclusions

NASH has become a global pandemic and the incidence of NASH-associated cirrhosis and development of hepatocellular carcinoma has been increasing [65,66]. Currently, there are no FDA-approved therapies to treat NASH. In the PIVENS (Pioglitazone, Vitamin E, or Placebo for Nonalcoholic Steatohepatitis) clinical trial neither vitamin E nor pioglitazone [67] reversed hepatic fibrosis. Furthermore, vitamin E can increase the risk of bleeding and has not been recommended for those with diabetes, and pioglitazone therapy can increase weight, potentially being counterproductive in those with obesity and metabolic syndrome. Proglumide is an older drug that was originally developed 30 years ago for peptic ulcer disease, but commercialization was halted with the development of more potent proton pump inhibitors. However, proglumide has been tested in over 600 human subjects where it was found to have a broad safety profile and without significant toxicity. A Phase 1 clinical trial (NCT04152473) in human subjects with NASH recently demonstrated the broad safety profile of this compound, and a pharmacokinetic study (NCT04814602) showed similar uptake and excretion in those with cirrhosis compared to healthy controls. Future studies are planned to test its efficacy in reversing histologic NASH in human subjects.

New strategies are needed to tackle the problem of NASH with safe and effective compounds. Proglumide shows potential in this current study by improving hepatic histology and decreasing fibrosis in mice with NASH through several pathways including inhibition of the CCK receptor, serving as a partial agonist at the FXR, and rendering the gut microbiome less hepatotoxic. In conclusion, our investigation demonstrates novel pathways that the CCK receptor inhibitor proglumide may act to improve NASH.

## 6. Patents

Georgetown University has a pending patent application for the use of proglumide in liver diseases.

## Figures and Tables

**Table 1:** Effects of CDE diet and proglumide on mouse livers compared to controls.

Treatment Group	AST, U/L	ALT, U/L	T. Bili, U/L	Inflammation Score	Fibrosis Score	Steatosis Score
1. Control	139 ± 19.7	60.2 ± 7.6	0.36 ±0.05	0	0	0
2. CDE/ Reg	420 ± 63	196 ± 42	1.08 ± 0.13	1.6 ± 0.17	1.13 ± 0.15	4.26 ± 0.15
3. CDE/Prog	248 ± 28.7 *	116 ± 15.2 *	0.44 ± 0.03 *	0.26 ± 0.11 *	0.53 ± 0.13 *	2.8 ± 0.26 *
* *p*-value 2 vs. 3	*p* = 0.01	*p* = 0.04	*p* < 0.0001	*p* = 0.017	*p* < 0.001	*p* = 0.02

AST: aspartate transferase; ALT: alanine transferase; T. Bili: total bilirubin; CDE/Reg: choline deficient diet with ethionine and regular water; CDE/Prog: choline deficient diet with ethionine with proglumide treated water. Control: mice fed standard chow and untreated water.

## Data Availability

The datasets used and analyzed during the current study are available upon request.

## Supplementary Materials

The following supporting information can be downloaded at: https://www.mdpi.com/article/10.3390/ijms23031899/s1.
