# Supplementary material for: Proglumide Reverses Nonalcoholic Steatohepatitis by Interaction with the Farnesoid X Receptor and Altering the Microbiome
Source: Int J Mol Sci. 2022 Feb 8;23(3):1899. doi: 10.3390/ijms23031899 (PMC8836891; doi:10.3390/ijms23031899)
Supplement: Supplementary file 1 [file ijms-23-01899-s001.zip › ijms-1557976-supplementary.pdf]

Supplemental Materials

Table S1. PCR primers used to measure DEGs

**Gene**	**Forward 5'- 3'**	**Reverse 5' – 3'**
CK17	ACCCTCCCGAGATTACAACC	CAAGGCGTGTTCTGTCTCAA
Collagen 1α	CGCCATCAAGGTCTACTG	ACGGGAATCCATCGGTC
Collagen 4	GATGGGCTATCCTGGAACCACT	TCTCTCCTCGTTCGCCTTTGG
TGFβR2	TTTCGGAAGAATACACCAC	GACACGGTAGCAGTAGAA

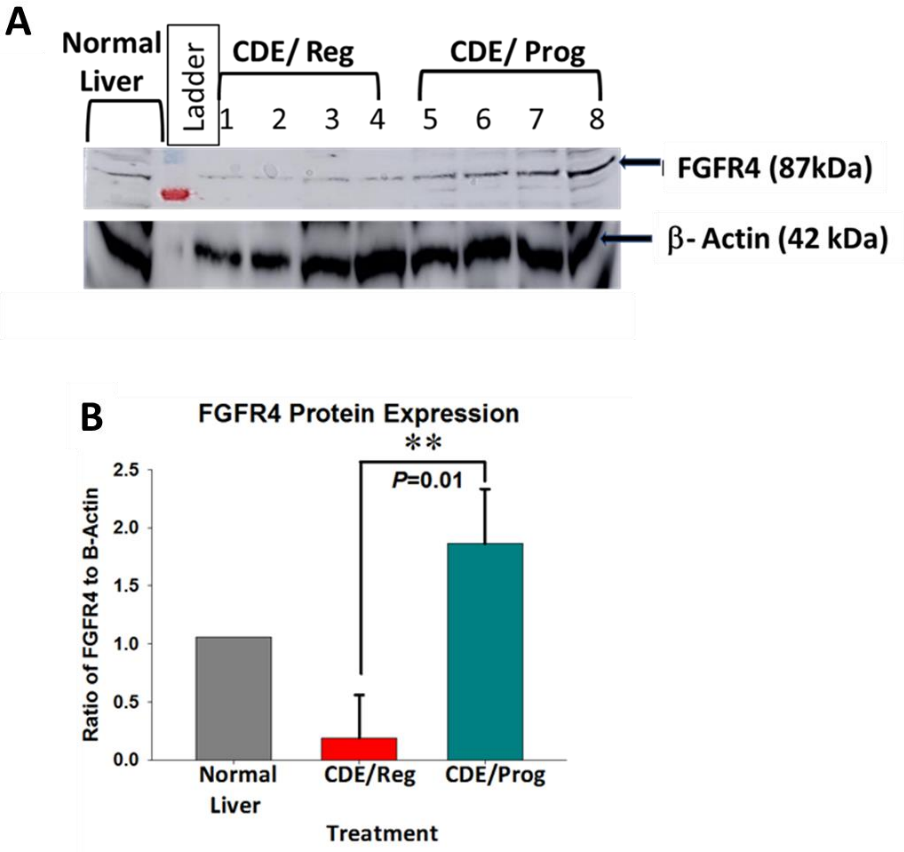

Figure S1. (A) FGFR4 protein expression by western blot is shown for NASH livers of mice on CDE/Reg diet and from livers of mice on CDE/Prog diet compared to mice on control diet (normal liver). Protein expression is normalized with β-actin. (B) Densitometry analysis of the western blot above for FGFR4 protein expression is analyzed and plotted as a ratio normalized to β-actin. FGFR4 expression is significantly increased in the mice on the CDE diet treated with proglumide compared to mice on the CDE diet with untreated water (**P=0.01).

Supplemental Materials

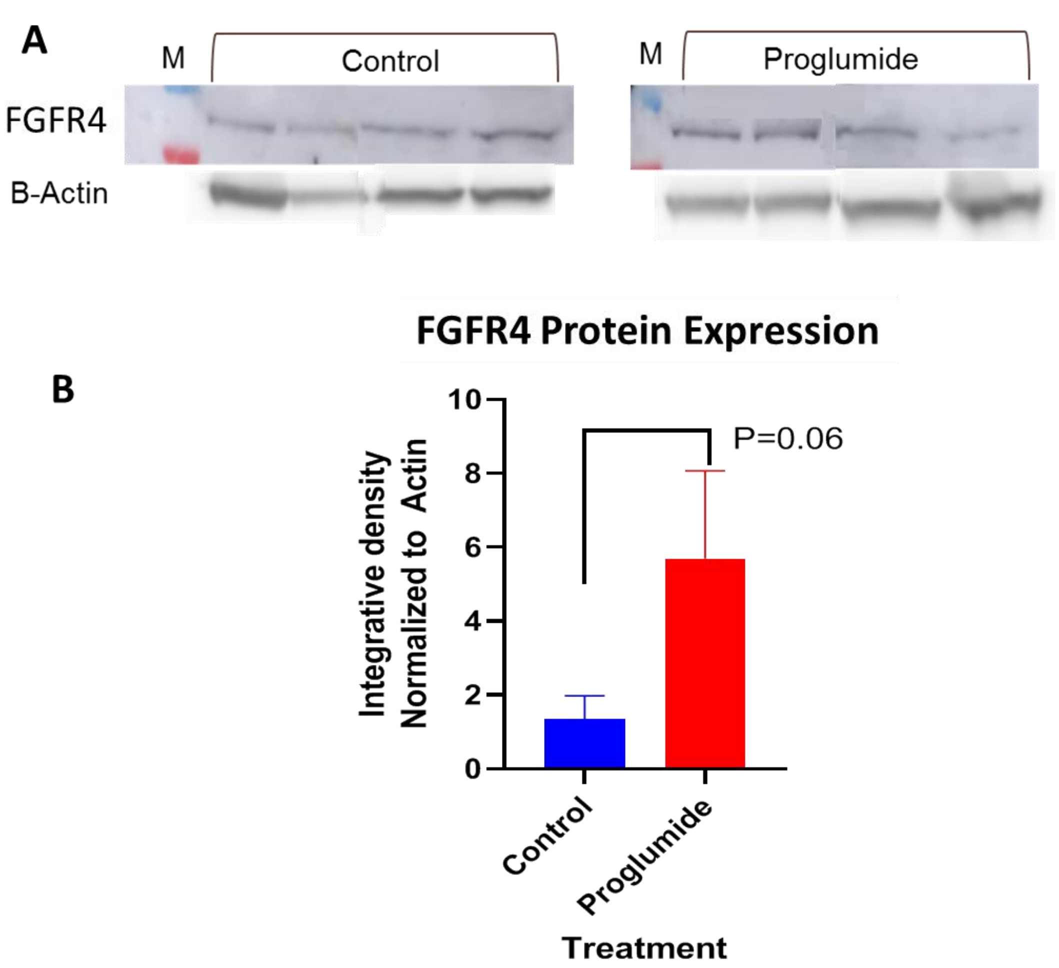

Figure S2. FGFR4 expression in the intestine of mice fed standard chow with or without proglumide in the drinking water. (*A*) Western blot of FGFR4 protein expression in Control mice on the left and in mice treated with proglumide on the right. (*B*) Densitometry analysis of western blot shows increased FGFR4 protein expression in the intestines of proglumide treated mice is 3-fold higher than controls (P=0.06).
